# Novelty and Priority in Medicine

**Published:** 1887-12

**Authors:** 


### EDITORIAL.

### NOVELTY AND PRIORITY IN MEDICINE.

The desire to discover and to present to the profession some novelty, and to secure credit for priority in its introduction, seems not to have abated in the last few years, if one may judge of it by recent observation. This fondness for what some call *enterprise* is not confined to the commercial world, for we see around us many evidences that the medical profession is not exempt from it. The odor of sulphureted hydrogen has scarcely left either patient or physician, since the recent explosion of the so-called *Bergeon's Method* of treatment of phthisis, before an equally unfounded claim seems to have been put forth for a rival for cocaine, which was represented as possessing greater anæsthetic and mydriatic properties than cocaine or atropine. Regarding this new local anæsthetic, known as Gleditschine or Stenocarpine, we are now informed that careful analysis has been made of a specimen of this new alkaloid, "which is derived from *Gleditschia Triacanthos*," and that the result has been that it "contains 6 per cent. of cocaine, and a sulphate of a salt, which further experiment is likely to prove to be Atropia." Also, that after careful experiment with the leaves of *Gleditschia Triacanthos* it was found "that they contain only an infinitesimal percentage of an amorphous alkaloid, devoid of anæsthetic or mydriatic properties."

Mr. F. A. Thompson, who conducted these experiments in the laboratory of Messrs. Parke, Davis & Company, at Detroit, says: "In the light of these facts it seems probable that the Stenocarpine sensation should be classed with the Hopeine fraud of malodorous memory, and that the physicians who have already published reports regarding Gleditschine, or Stenocarpine, have been the victims of a clever hoax."

Thus is afforded further evidence of the desirability of trusting to tried remedies that have stood the test of time—the most reliable of all tests.

If, as a result of these unfounded claims, more caution shall result in accepting the assurances given, without fair trial or reliable data, regarding every alleged novelty introduced into medicine, and lead to better use of the remedies, whose value time and experience have demonstrated, instead of seeking somthing new or unusual for the sake of novelty, these deceptions, like their predecessors, will not have failed to contribute to a good end.

